# Randomized, Multicenter Study of Gefitinib Dose-escalation in Advanced Non-small-cell Lung Cancer Patients Achieved Stable Disease after One-month Gefitinib Treatment

**DOI:** 10.1038/srep10648

**Published:** 2015-07-28

**Authors:** Cong Xue, Shaodong Hong, Ning Li, Weineng Feng, Jun Jia, Jiewen Peng, Daren Lin, Xiaolong Cao, Siyang Wang, Weimin Zhang, Hongyu Zhang, Wei Dong, Li Zhang

**Affiliations:** 1 State Key Laboratory of Oncology in South China, Collaborative Innovation Center for Cancer Medicine, Department of Medical Oncology, Sun Yat-Sen University Cancer Center, Guangzhou, China; 2 Department of Oncology, Guangzhou Chest Hospital, Guangzhou, China; 3 Department of Medical Oncology, the First People’s Hospital of Foshan, Foshan, China; 4 Department of Medical Oncology, Dongguan People’s Hospital, Dongguan, China; 5 Cancer Center, Zhongshan People’s Hospital, Zhongshan, China; 6 Department of Oncology, Jiangmen Central Hospital, Jiangmen, China; 7 Department of Oncology, Panyu District Central Hospital, Guangzhou, China; 8 Department of Oncology, the Fifth Affiliated Hospital of Sun Yat-sen University, Zhuhai, China; 9 Department of Oncology, General Hospital of Guangzhou Military Command of PLA, Guangzhou, China; 10 Department of Hematology and Rheumatism, the First People’s Hospital of Shunde, Foshan, China

## Abstract

There is no consensus on the optimal treatment for patients with advanced non-small-cell lung cancer (NSCLC) and stable disease (SD) after gefitinib therapy. This randomized, open-label, multicenter study aimed to explore whether dose-escalation of gefitinib would improve response and survival in NSCLC patients who achieved SD after one-month of standard gefitinib dosage. Between May 2009 and January 2012, 466 patients were enrolled and 100 eligible patients were randomized (1:1) to receive either a higher dose (500 mg/d; H group) or to continue standard dose (250 mg/d; S group) of gefitinib. Objective response rate (ORR) was similar between the two groups (12.5% vs 12.5%, *p* = 1.000). There were no significant differences regarding progression-free survival (PFS) and overall survival (OS) between both arms (H group vs S group: median PFS, 5.30 months vs 6.23 months, *p* = 0.167; median OS, 13.70 months vs 18.87 months, *p* = 0.156). Therefore, dose-escalation of gefitinib does not confer a response or survival advantage in patients who achieve SD with one month of standard-dose gefitinib treatment.

Non-small-cell lung cancer (NSCLC) is a leading cause of cancer mortality worldwide^1^. Although chemotherapy remains the mainstream treatment of NSCLC, the emergence of targeted therapy has led to tremendous advancement. Epidermal growth factor receptor (EGFR) mutations play an important role in driving the growth of NSCLC. The most commonly detected drug-sensitive mutations are deletions in exon 19 and point mutations in exon 21. EGFR mutations have been confirmed as a predictive biomarker for EGFR tyrosine kinase inhibitors (TKIs) treatment in several clinical trials^2^,^3^,^4^,^5^.

Currently, gefitinib and erlotinib are approved for the first-line treatment of advanced NSCLC with sensitive EGFR mutations. However, the routine clinical dose of gefitinib (250 mg/day) is only one-third of its maximum tolerated dose (MTD) while the dose of erlotinib (150 mg/day) is at its MTD^6^,^7^. Until now, no head-to-head trials have been available to determine whether erlotinib is superior to gefitinib in terms of response rate or survival. Therefore, dose escalation of gefitinib is feasible and warrants further investigation. Some previous studies have showed that higher drug exposure of gefitinib was associated with better clinical outcomes, especially in patients with wild type EGFR^8^,^9^. However, other studies found that gefitinib dose-escalation did not show superior efficacy in nonselective population^10^,^11^. We assume that a portion of specific patients may benefit from higher drug dosage. Kaira *et al.* analyzed eleven published reports about the effectiveness of erlotinib re-challenge (may be considered as an EGFR-TKI dose-escalation) after previous gefitinib failure in advanced NSCLC patients and revealed that patients who had stable disease (SD) but not those with a complete response (CR) plus partial response (PR) or progressive disease (PD) for gefitinib benefited from erlotinib. Thus, the patients who achieved CR+PR or PD with gefitinib were either most sensitive or primarily resistant to initial EGFR-TKIs. Those who achieved SD may be the target patients who will benefit from higher dose EGFR TKIs^12^. Currently, no consensus has been reached on the optimal treatment of these SD patients. Whether these patients should switch to other anti-cancer drugs or have more intensive dose of initial drugs to achieve PR or CR remains controversial. A phase II trials assessing chemotherapy continuation vs switch in advanced NSCLC patients with SD after two course of chemotherapy found no difference in response rates^13^. However, no studies have investigated the efficacy of gefitinib dose-escalation in these SD patients after one month of standard gefitinib dosage.

Based on previous studies, we postulated that gefitinib drug escalation of gefitinib may improve response and survival in NSCLC patients who achieved SD after one month of standard gefitinib dosage.

## Results

### Patients

**Figure 1:**
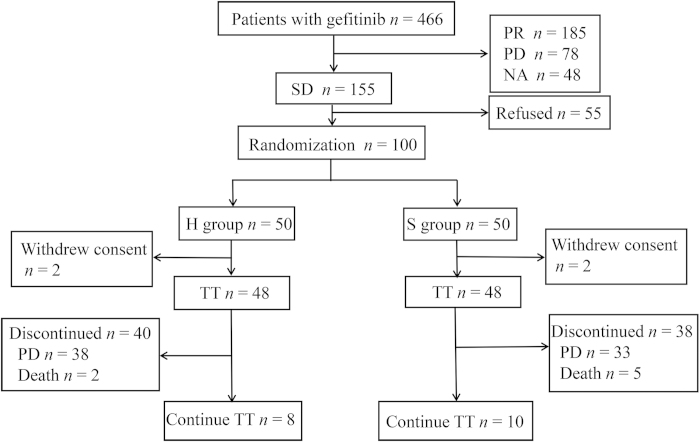
Consort diagram of the disposition of participants. PR, partal response; PD, progressive disease; NA, unevaluable; SD, stable disease; TT, trial treatment.

Between May 2009 and January 2012, 466 patients treated with gefitinib (250 mg/d) were enrolled from 10 centers in China. After one month, patients who achieved PR (*n* = 185), PD (*n* = 78) or cannot be evaluated (*n* = 48) were excluded. Among those who achieved SD (*n* = 155), 55 patients refused to participate in the study. Finally, a total of 100 patients were randomly assigned (1:1) to receive either higher dose (500 mg/d, H group) or continue standard dose (250 mg/d, S group) of gefitinib (Fig. 1). Four patients withdrew consent after randomization (not received protocol treatment), leaving 48 patients in each arm. Between-arm baseline characteristics were mostly balanced (Table 1). Among 96 intention-to-treat (ITT) patients, 53 (55%) were women, 63 (66%) were never-smokers, 85 (89%) had adenocarcinoma. Median follow-up duration was 12.73 months. On the day of data cut-off (Jun 20, 2014), 8 patients from H group and 10 patients from S group remained on gefitinib treatment. Median treatment duration was 4.40 months in H group and 5.40 months in S group.

A total of 83 patients (86.5%) gave their consent for biomarker analyses. Fifty-one of them provided tumor samples and the remaining 32 patients provided blood samples. The overall EGFR mutation rate was 30.1% (25/83). However, in the blood samples, only 1 patient was found to harbor EGFR mutation (3.1%) while the EGFR mutation rate tested in the tumor tissue was 47.1% (24/51).

### Efficacy

**Figure 2:**
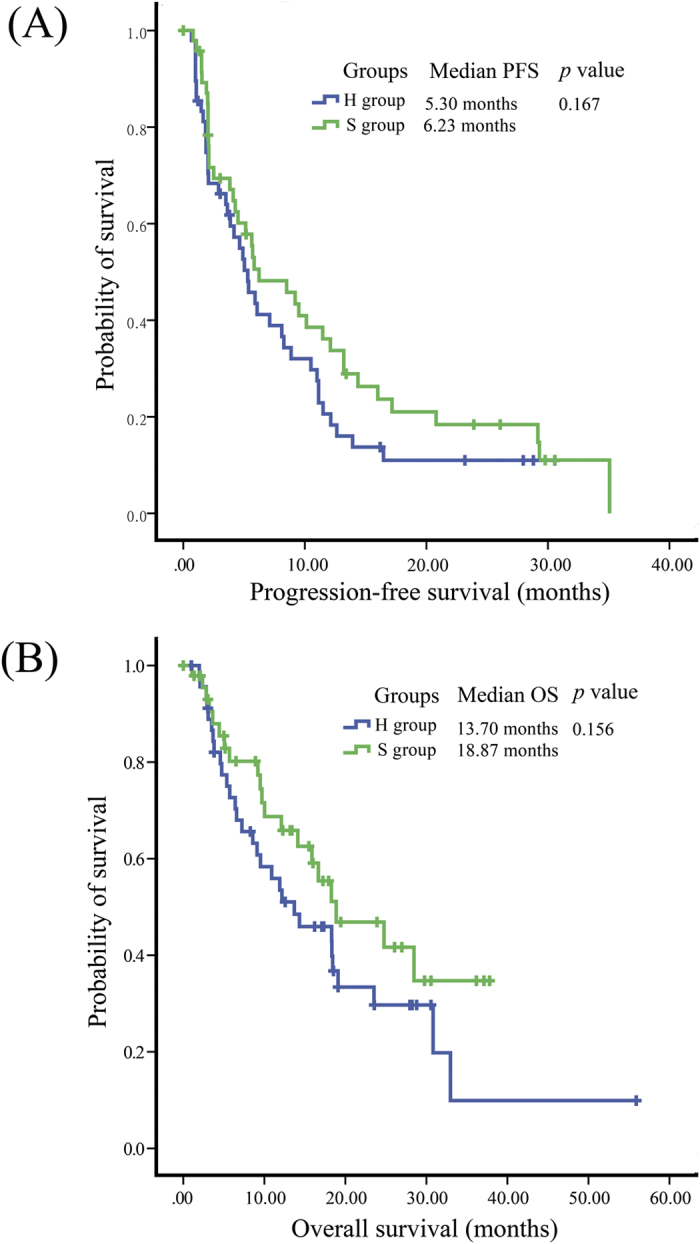
Kaplan–Meier estimates of (**A**) progresion-free survival (PFS) and (**B**) overall survival (OS) in patients who received 500 mg/d (H group) or 250 mg/d (S group) gefitinib after achieving stable disease after one-month 250 mg/d gefitinib.

In the ITT analysis, the objective response rates (ORRs) were both 12.5% in the two arms (*p* = 1.000): no CR occurred and 6 patients experienced PR in each group. Twenty-eight patients (58.3%) from H group and 29 patients (60.4%) from S group had SD. Fourteen patients (29.2%) in H group and 13 patients (27.1%) in S group had PD. No significant differences were observed regarding median progression-free survival (PFS) (5.30 months vs 6.23 months, *p* = 0.167;) or median overall survival (OS) (H group vs S group, 13.70 months vs 18.87 months, *p* = 0.156) between the two groups (Fig. 2).

**Figure 3:**
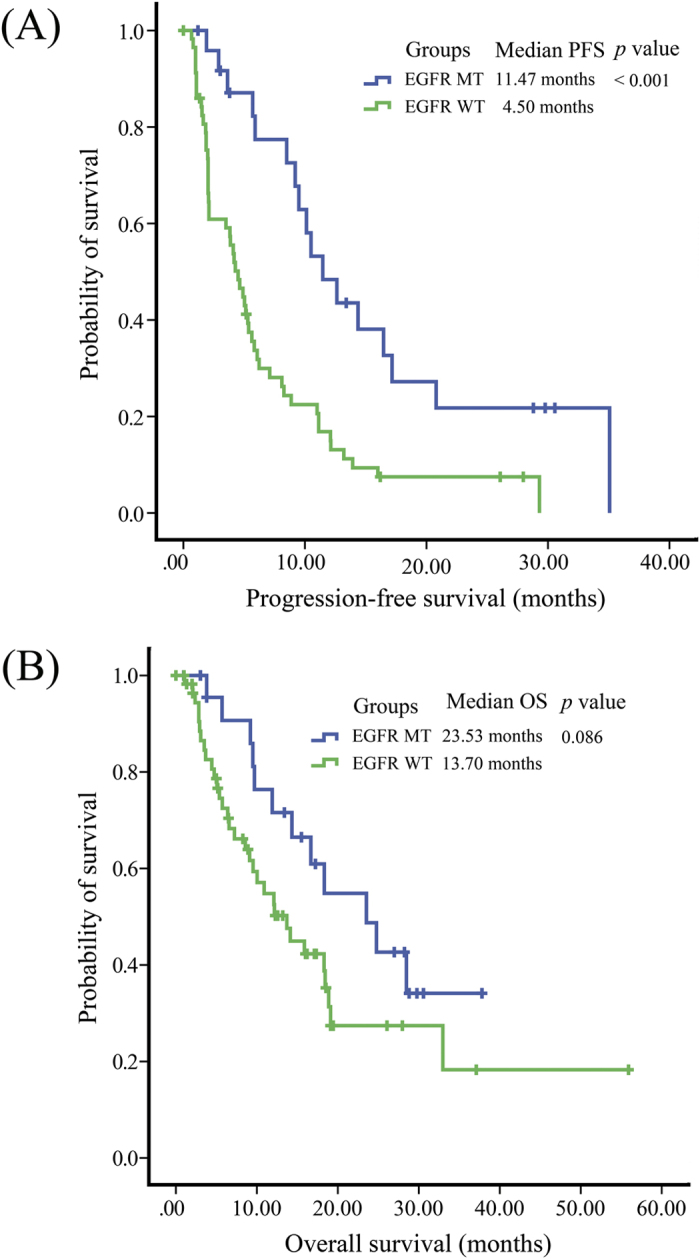
Kaplan–Meier estimates of (**A**) progression-free survival (PFS) and (**B**) overall survival (OS) in NSCLC patients, stratified by EGFR mutations status.

**Figure 4:**
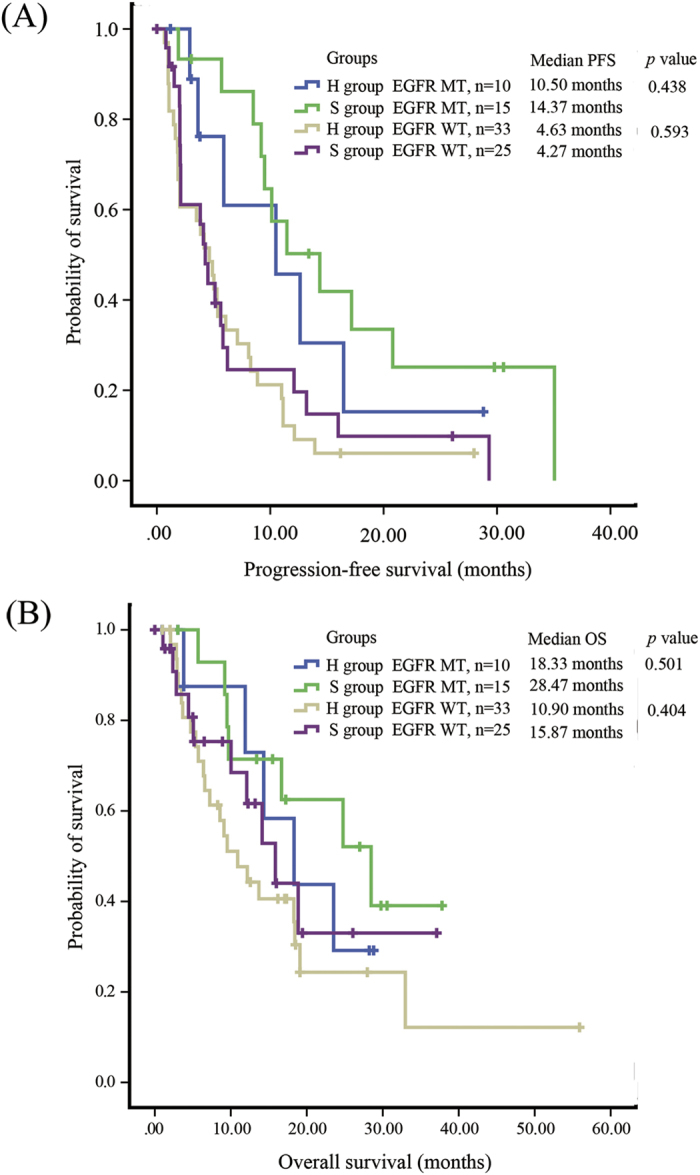
Kaplan–Meier estimates of (**A**) progression-free survival (PFS) and (**B**) overall survival (OS) in NSCLC in patients who received 500 mg/d (H group) or 250 mg/d (S group) gefitinib after achieving stable disease after one month of 250 mg/d gefitinib, stratified by EGFR mutations status.

In 83 consented patients with known EGFR mutation status (25 mutant (MT) EGFR and 58 wild type (WT) EGFR), PFS was significantly longer in patients with MT EGFR than those with WT EGFR (median PFS: 11.47 months vs 4.50 months, *p* < 0.001). Difference was also found in OS with similar tendency (median OS: 23.53 months vs 13.70 months, *p*  = 0.086) (Fig. 3). Patients with MT EGFR has higher ORR than those with WT EGFR, although no statistical significance was reached (5/25 vs 6/58, *p* = 0.242). We then stratified H group and S group by EGFR mutation status. In MT EGFR patients, no significant difference in PFS (*p* = 0.438) or OS (*p* = 0.501) was observed between H group and S group (Fig. 4). ORR was also similar between experiment and control groups (1/10 vs 4/15, *p* = 0.615). In WT EGFR patients, PFS and OS were also similar between H group and S group (*p* = 0.593 and 0.404, respectively) (Fig. 4). No significant ORR difference was observed between the two groups in WT EGFR patients (5/33 vs 1/25, *p* = 0.222).

Regarding the predictive value of rash severity one month after randomization (first evaluation of tumor response), we found that patients with higher grade rash (≥ grade 2, *n* = 20) had longer PFS than those with lower grade (≤ grade 1, *n* = 76) (8.27 months vs 5.3 months; *p* = 0.046). Also severer rash at first evaluation was significantly associated with better response (ORR, 25% vs 9.2%; p = 0.050). But in multivariate analysis adjusted for EGFR mutation status, gender and smoking status, rash severity at first month failed to predict PFS (*p* = 0.062) or response rate (*p* = 0.057) superiority.

### Safety

The most common adverse events (AEs) are listed in Table 2. The most commonly reported AEs included acne-like rash, diarrhea and abnormal aminotransferases. Forty-one out of 48 (85.4%) patients in H group and 33 out of 48 (68.8%) patients in S group had rash events (*p* = 0.057). Seven out of 48 (14.6%) patients in H group and none of 48 in S group had grade 3/4 acne-like rash (*p* = 0.006). No severe adverse events were observed. Two deaths in H group and five in S group were attributed to disease progression.

### Plasma gefitinib concentration

**Figure 5:**
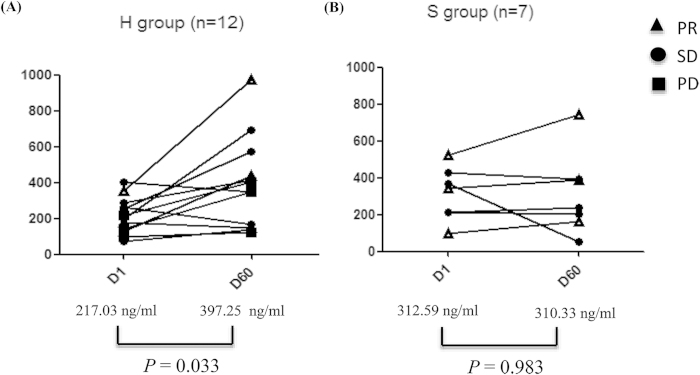
Plasma gefitinib concentration at randomization and 60 days thereafter in patients who received 500 mg/d gefitinib or 250 mg/d gefitinib. PR, partal response; PD, progressive disease; SD, stable disease.

A total of 19 (19.4%, 12 from H group and 7 from the S group) paired samples were available. Mean plasma gefitinib concentration between H group and S group at baseline (D1) was comparable (217.03 vs 312.59 ng/ml, *p* = 0.109). Mean plasma gefitinib concentration raised 60 days after randomization (D60) in H group (D1 vs D60, 217.03 vs 397.25 ng/ml, *p* = 0.033) but remained stable in S group (D1 vs D60, 312.59 vs 310.33 ng/ml, *p* = 0.983) (Fig. 5).

## Discussion

Although EGFR mutations have been acknowledged as the most important predictor for the clinical outcome of patients receiving EGFR TKIs, the role of drug exposure remains controversial. In addition to our previous study^9^, Nakamura *et al.* also assessed the relationship between the plasma gefitinib concentration and its efficacy in patients with advanced NSCLC. In subgroup analysis, median PFS was similar between EGFR MT patients and WT patients in the high D8/D3 group, whereas the WT EGFR patients in the low D8/D3 group showed the worst median PFS^8^. Nakamura *et al.* concluded that better survival was correlated with a higher drug plasma concentration in WT EGFR patients. However, in two randomized phase II trials (IDEAL 1 and 2), gefitinib 250 mg/d was as effective as, and better tolerated than, gefitinib 500 mg/d^10,11^. Moreover, Hirano *et al.* found that the Cmax of gefitinib in patients with PR was significantly lower than that of patients with SD (median Cmax: 278 vs 588 ng/mL, respectively; *p* < 0.05). A significant negative correlation was found between the area under the plasma concentration time curve (0 -24 h) of gefitinib and survival (*p* < 0.05)^14^. They concluded that a high plasma concentration of gefitinib does not guarantee long-term therapeutic effects in MT EGFR patients.

The current study describes the unique multicenter randomized study that explores the hypothesis that better treatment outcome of EGFR TKIs could also be attributed, in part, to higher drug exposure in patients with advanced NSCLC. Our study differed from the IDEAL 1 and 2 studies. In IDEAL 1 and 2, the patients were randomly assigned two dose levels of gefitinib (250 versus 500 mg/d) at treatment initiation, whereas only patients who achieved SD after one month of treatment with 250 mg/d gefitinib were randomly assigned to 250 mg/d versus 500 mg/d of gefitinib in the present study. The present study is likely to enrich a more homogeneous group of patients, is expected to minimize the dilution effect, and may maintain practical and feasible patients’ accrual levels. In our study, gefitinib dose escalation significantly increased the drug plasma concentration. However, this increase did not translate into the improvements in patient’s response or survival. The result was consistent with those in previous studies showing that 500 mg gefitinib did not provide additional benefits. Patient selection provides one possible explanation for this. It may be that only a specific population benefits from dose escalation, but in the current study, the clinical characteristics of “SD after one-month gefitinib treatment” were insufficient to select these patients from the entire population. In another study by Yeo *et al.*, a decrease dose of erlotinib from 150 mg/d to 25 mg/d (equivalent to gefitinib 250 mg/d) in MT EGFR NSCLC patients showed a similar response rate but fewer side-effects^15^. Therefore, erlotinib 25 mg/d could be an alternative dosing scheme. Taken together, these results imply that gefitinib 250 mg/d is currently the lowest and clinically active dose in SD patients and that a dose-escalation of EGFR-TKIs within the MTD might not improve clinical outcomes.

Whether skin rash predicted the outcome of patients treated with EGFR TKI remains controversial. In our study, patients were grouped into those presenting ≥ grade 2 rash and those presenting grade 0-1 rash. Although patients with severer rash tended to have longer PFS, this was not significant via multivariate analysis. Few patients in our study had ≥ grade 2 rash even in the H group, a finding that was consistent with that in the study by Rukazenkov *et al.* They found that gefitinib was significantly accumulated in tumor tissue; thus, the plasma concentration was lower than that of erlotinib, providing the concentration needed at its target to achieve effective EGFR inhibition in the tumor while causing less skin toxicity than erlotinib^7^.

There were several limitations in the present study. Firstly, because it was not a double-blinded trial, there may be bias in the evaluation of efficacy and toxicity. Secondly, caution should be exerted regarding the small number of patients in our study. Thirdly, the enrollment was based on the clinical response and not the EGFR mutation status; thus, the patient selection was suboptimal. Nevertheless the clinicopathologic characteristics of the two groups were balanced in two groups and we believe the results are still reliable.

In summary, gefitinib 500 mg/d does not confer a response or survival advantage over gefitinib 250 mg/d in patients with advanced NSCLC who achieved SD with one-month of gefitinib 250 mg/d. Given the cost-effect ratio and safety concerns, routine application of gefitinib dose-escalation is not recommended in such a population.

## Patients and Methods

### Patient eligibility

Eligibility criteria included histological or cytological confirmation of advanced NSCLC; recurrent or refractory disease after at least one previous regimens; achievement of SD after one-month of gefitinib 250 mg/d therapy; measurable lesions defined by Response Evaluation Criteria in Solid Tumors (RECIST)^16^; Eastern Cooperative Oncology Group (ECOG) performance status (PS) of 0 to 2; and satisfactory renal, hematological and cardiac function. Major exclusion criteria included previous EGFR-TKIs therapy, pregnancy, breast-feeding or unable to take oral medications. Patients with stable brain metastasis (BM) were allowed.

### Study design

This open-label, randomized, multi-center study recruited patients from 10 centers in China. Eligible patients were randomly assigned to receive gefitinib at 250 mg/d (S group) or 500 mg/d (H group). Gefitinib was administered orally in both groups. Patients would continue treatment until disease progression or intolerable toxicity. The choice of post-progression treatment was left to the investigators. Dose modification was not allowed in this trial.

Objective tumor response was evaluated by blinded radiologists using RECIST every 4 weeks until progression. The patients were followed for survival every 3 months and for safety 30 days after the last study drug administration. Adverse events (AEs) were evaluated according to the National Cancer Institute Common Terminology Criteria for Adverse Events version 3.0 (NCI-CTCAE 3.0). Since rash was one of the most recognized AEs of EGFR-TKIs and may be a surrogate of drug exposure, we tried to investigate the association between rash severity and prognosis. Rash events were recorded one month after randomization (at the time of first radiographic evaluation). And patients were grouped into those presenting with rash severity ≥ grade 2 and those with grade 0-1, but excluding patients who progressed or did not follow-up before 1 month. The study was carried out in accordance with the approved guidelines and regulations including the Declaration of Helsinki. The study protocol was approved by the institutional ethics review board, and informed consent was obtained for each patient. This trial was registered in clinicaltrials.gov (ClinicalTrials.gov number: NCT01017679).

### EGFR mutation tests

Paraffin-embedded specimens were collected for EGFR mutation tests using direct DNA sequencing (plenty of tissue) or PCR (limited tissue) with the method in patients who provided tumor tissue^17^. In case tumor tissue was unavailable, plasma samples were collected for EGFR mutation tests using denaturing high-performance liquid chromatography (DHPLC) with patient consent^18^.

### Plasma gefitinib concentration analysis

Blood samples were collected in consenting patients on Day 1 (randomization) and Day 60 (first evaluation). They were centrifuged at 3,000 rpm, 4 °C for 10 minutes. The plasma samples were then stored at −80 °C. The plasma gefitinib concentration was analyzed using high-performance liquid chromatographic-tandem mass spectrometric method as previously described^19^.

### Statistical analysis

We used a response rate of 5% (observed using the standard dose of gefitinib in WT EGFR NSCLC) as the null hypothesis. To assume a 10% improvement in the response rate with the proposed regimen, at a significance level of 5% and a statistical power of 80%, we determined the sample size to be 100.

The primary endpoint was the objective response rate (ORR). Secondary endpoints were progression-free survival (PFS), overall survival (OS) and the risk of common AEs. PFS was defined as the duration between the date of randomization and the date of disease progression, death from any cause or the last follow-up. OS was defined as the duration from the date of randomization to the date of death from any cause or last follow-up. The ORR and risk of AEs were evaluated using Chi-square test or Fisher’s exact test. Quantitative variables were compared with Student’s t test or Wilcoxon’s test for unpaired series when the variables were not normally distributed. PFS and OS were estimated using Kaplan-Meier methodology. The log-rank test was used to compare survival between the two treatment groups. All of the statistical tests were performed using SPSS (Statistical Package for the Social Sciences) version 21.0 software (SPSS, Chicago, IL). A two-tailed *p* value of less than 0.05 was deemed significant.

## Additional Information

**How to cite this article**: Xue, C. *et al.* Randomized, Multicenter Study of Gefitinib Dose-escalation in Advanced Non-small-cell Lung Cancer Patients Achieved Stable Disease after One-month Gefitinib Treatment. *Sci. Rep.* **5**, 10648; doi: 10.1038/srep10648 (2015).

## Figures and Tables

**Table 1:** Baseline characteristics of the included patients.

**Variables**	**H group (*n* = 48)**	**S group (*n* = 48)**
Median age in years (range)	55.5 (33-83)	57.5 (32-83)
Gender		
Male	24	19
Female	24	29
Current or former smoker, n	18	15
Histopathology, n		
Adenocarcinoma	44	41
Non-adenocarcinoma	4	7
EGFR status*, n		
Mutant type (MT)	10	15
Wild type (WT)	33	25
Unknown	5	8
Current treatment, n		
Second- or third-line	43	39
Fourth-line	2	3
First-line or maintenance	3	6
Former platinum-based chemotherapy, n	42	42

^*^Methods: PCR or direct sequencing (formalin-fixed paraffin embedded tissue, *n* = 51) or HPLC (blood sample, *n* = 32); H groups refers to escalated gefitinib (500 mg/d), while S group refers to standard-dose gefitinib (250 mg/d).

**Table 2:** Incidence of common adverse events associated with the two dose regimens of gefitinib.

**Types of adverse events**	**H group (*n* = 48)**		**S group (*n* = 48)**		***P* value**	
	**All Grade**	**Grade 3-4**	**All Grade**	**Grade 3-4**	**All Grade**	**Grade 3-4**
Acne-like rash, n (%)	41 (85.4%)	7 (14.6%)*	33 (68.8%)	0	0.567	0.012*
Diarrhea, n (%)	8 (16.7%)	0	6 (12.5%)	0	0.577	NA
Abnormal aminotransferases level, n (%)	3 (6.3%)	0	1 (2.1%)	0	0.242*	NA

^*^Fisher exact test.

H groups refers to escalated gefitinib (500 mg/d), while S group refers to standard dose gefitinib (250 mg/d). NA, not applicable.
